# New Biocide Foam Containing Hydrogen Peroxide for the Decontamination of Vertical Surface Contaminated With *Bacillus thuringiensis* Spores

**DOI:** 10.3389/fmicb.2018.02295

**Published:** 2018-09-27

**Authors:** Esther Le Toquin, Sylvain Faure, Nicole Orange, Fabienne Gas

**Affiliations:** ^1^Laboratoire Innovations technologiques pour la Détection et le Diagnostic, Service de Pharmacologie et Immunoanalyse, DRF, CEA, INRA, Bagnols-sur-Cèze, France; ^2^Laboratoire de Microbiologie Signaux et Microenvironnement, Université de Rouen, Evreux, France; ^3^Laboratoire des Procédés Supercritiques et Décontamination, Service d’études des technologies pour l’assainissement démantèlement et l’étanchéité, Univ. Montpellier, DEN, CEA, Bagnols-sur-Cèze, France

**Keywords:** *Bacillus thuringiensis*, spores, decontamination, hydrogen peroxide, foam, biocide

## Abstract

Despite scientific advances, bacterial spores remain a major preoccupation in many different fields, such as the hospital, food, and CBRN-E Defense sector. Although many disinfectant technologies exist, there is a lack for the decontamination of difficult to access areas, outdoor sites, or large interior volumes. This study evaluates the decontamination efficiency of an aqueous foam containing hydrogen peroxide, with the efficiency of disinfectant in the liquid form on vertical surfaces contaminated by *Bacillus thurengiensis* spores. The decontamination efficiency impact of the surfactant and stabilizer agents in the foam and liquid forms was evaluated. No interferences were observed with these two chemical additives. Our results indicate that the decontamination kinetics of both foam and liquid forms are similar. In addition, while the foam form was as efficient as the liquid solution at 4°C, it was even more so at 30°C. The foam decontamination reaction follows the Arrhenius law, which enables the decontamination kinetic to be predicted with the temperature. Moreover, the foam process used via spraying or filling is more attractive due to the generation of lower quantity of liquid effluents. Our findings highlight the greater suitability of foam to decontaminate difficult to access and high volume facilities compared to liquid solutions.

## Introduction

Certain Gram-positive bacteria, like *Bacillus* and *Clostridium* species, are able to protect themselves from environmental stress or nutrient depletion by a sporulation process. Vegetative cells that pass into a dormant spore state are known to be much more resistant to the environment and to disinfection treatments than growing cells of the same species (Nicholson et al., 2000; Driks, 2002b; Piggot and Hilbert, 2004; Higgins and Dworkin, 2012; Wood et al., 2015). Several factors have been identified as responsible for the increased spore resistance. A thick spore coat protects the inner layers from lytic enzymes and from many chemicals, including chlorine dioxide and sodium hypochlorite (Driks, 1999, 2002a). The extremely low spore permeability enables the protection of the inner spore membrane against hydrophilic agents and possible UV and γ-radiation (Riesenman and Nicholson, 2000; Doona et al., 2015). The reduced water content in the core protects the spore from dry and wet heat, enabling it to survive for longer periods of time in a hostile environment (Setlow, 2006; Leggett et al., 2012). The spore DNA is saturated with small acid-soluble proteins (SAPS) of the α/β type and the high presence of calcium-dipicolinic acid chelate inside the spore core makes spore destruction more difficult by UV radiation, heat, and some genotoxic chemicals (Setlow et al., 2006; Moeller et al., 2009). Finally, the DNA repair mechanisms occur just at the beginning of germination when the spore returns to growth-cell life (Setlow, 1992). Bacterial spores have always been considered as a threat either through their potential for use as biological weapons (e.g., the 2001 anthrax attack in the United States) (Schmitt and Zacchia, 2012), or because of food and hospital contaminations (Faille et al., 2014; Yi et al., 2016; Fernandes et al., 2017).

Several decontamination technologies have been proposed in the past and different sporicidal treatments are known. UV radiation and genotoxic chemicals damage spore DNA (Tennen et al., 2000); strong acid disrupts spore integrity; peroxynitrite and other chemicals damage the spore inner membrane (Genest et al., 2002), and heat or peroxides disrupt the spore germination apparatus by targeting key proteins (Loshon et al., 2001; Melly et al., 2002). The choice of a decontamination treatment depends on the way to use the technology, the biocide efficiency of the treatment, the type of contaminated surfaces, the energy quantity and basic materials needed for the decontamination, and the quantity of waste products after treatment (Sagripanti and Bonifacino, 1999; Wood et al., 2011; Ryan et al., 2014; van Bokhorst-van de Veen et al., 2015). Each treatment presents different advantages: different means of implementation for a method applying chemical products (wet wipes, sprays, or fumigation) (Otter and French, 2009; Kenters et al., 2017), no waste after treatment with UV radiation, good dispersion of biocide products by fumigation for closed areas. But few processes are able to efficiently decontaminate, without extra costs or high quantities of waste, outdoor, and indoor sites with large volumes or hard to access areas like ventilation pipes (Schmitt and Zacchia, 2012; Edmonds et al., 2014).

To date several laboratories offer commercial foaming solutions and sprayers to decontaminate surfaces. These disinfecting formulations contain foaming surfactants and an active substance, sodium hypochlorite or hydrogen peroxide, but not a stabilizing agent (Environmental Protection Agency, 2006). A stabilizing agent enables the foam to be stabilized in time, thus decreasing the foam drainage and increasing the contact time with the contamination (Dame et al., 2005). Foams with a controlled liquid fraction, containing a stabilizing agent and able to reduce spore contamination by six logs, have been successfully developed and patented in our laboratory (Faure et al., 2016). These foaming solutions are used with a dedicated foam generator that maintains the foam liquid fraction between 3 and 5%. These long-lifetime foams could be used to statically decontaminate outdoor and indoor facilities and equipment such as ventilation pipes, offices, cold-rooms, trains, and containers. Two ways of use were developed: sprayer application depositing a centimeter-thick layer of the decontamination foam on accessible contaminated surfaces, or a filling application for all volumes, even facilities difficult to access. Without treatment, all such places could become contamination exchange areas contributing to contamination propagation.

This study measured the disinfectant efficiency of a new biological decontamination foam using hydrogen peroxide as the disinfectant. The work was carried out on both foam and liquid (no air bubbles) forms to determine the decontamination efficiency. In addition, the study gave data related to the negative or positive contribution of each chemical product contained in the solution. For both foam and liquid forms, the decontamination impact from the stabilizer agent was evaluated. Further tests were performed by using the liquid form containing only the hydrogen peroxide. *Bacillus thuringiensis* (Bt) spores, biological indicators for decontamination tests in the field of CBRN Defense, were used for all tests. Moreover, to simulate difficult case of decontamination, all tests were performed on vertical surfaces contaminated with Bt spores. Different contact times and temperatures were tested for each decontamination technologies liquid and foam. Foam formulations with or without stabilizer agent were tested at three temperatures to study how the behavior of the foam is affected by temperature variations.

## Materials and Methods

### Bacterial Spore Preparation

*B. thuringiensis* DSM 5815 (provided by Leibniz-Institute DSMZ Co., Braunschweig, Germany) were used for this study, and prepared according to the protocol described by Wood et al. (2016). Briefly, frozen *B. thuringiensis* was inoculated into 10 mL of lysogeny broth media (LB, Becton Dickinson Co., Sparks, MD, United States) at 30°C for 16–18 h with continuous agitation at 160 rpm. Aliquots of 150 μl were then transferred into fresh media and incubated for 5 h at 30°C with continuous agitation at 160 rpm. The inoculum was spread onto a nutrient agar [0.5% (w/v) Tryptone, 0.3% (w/v) beef extract, 0.3% (w/v) NaCl, 2% (w/v) agar, and 0.01% (w/v) glucose], completed with 10 mL of metal solution [0.025% (w/v) MnSO_4_⋅H_2_O, 0.03% (w/v) CaCl_2_, 0.04% (w/v) (NH_4_)_2_SO_4_, 0.004% (w/v) MnCl_2_⋅4H_2_O, 0.0025% (w/v) CuSO_4_⋅5H_2_O, and 0.0025% (w/v) ZnSO_4_⋅7H_2_O] and incubated at 30°C. The sporulation progression in the petri dishes was checked by optical microscopy after 7–9 days. When the sporulation reached ≥95%, the spores were harvested from the plates using a rake and sterile distilled water, and washed four times in sterilized water by centrifugation and resuspension (4500 rpm at 4°C, two times 30 , 20 , and 15 min) to remove cell debris and media co-contaminants. The spore suspension was heat-treated at 70°C for 15 min with continuous agitation at 300 rpm. For heat-shock, the heat-treated spores were plunged into ice for 20 min and sonicated for 4 min at 45 Hz. The final titer of the suspension was determined by 1:10 and 1:5 serial dilutions, prepared using LB media and covered on a petri dish with appropriate solid growth media. The plates were incubated at 30°C overnight before CFU evaluation.

### Disinfectant Solutions

Five solutions were prepared with or without hydrogen peroxide at 12% (v/v) and with or without Xanthan 0.3% (w/v) (Sigma-Aldrich, St. Louis, Mo., United States), and Glucopon 215 UP commercial solution 1.1% (w/v) (BASF Canada Inc., Mississauga, Canada) respectively (**Table 1**). Solutions containing Glucopon 215UP (*S2, S3, S4*, and *S5* in **Table 1**) were tested both in the foam and in the liquid form. The solution *S1* was tested only as liquid. A static generator was used to mix air and foaming solutions to generate foam. A tube filled with glass balls generated the foam just before the coupon treatment. The generator parameters were set to obtain a foam with a liquid fraction between 3 and 6% (volume liquid/volume foam), with an average of 5.5%.

**Table 1:** Tested solutions formulated with Hydrogen peroxide, Xanthan, and Glucopon 215 UP.

Solution		Formula
*S1*	12%	Hydrogen peroxide
*S2*	12%	Hydrogen peroxide
	1.1%	Glucopon 215 UP
*S3*	12%	Hydrogen peroxide
	1.1%	Glucopon 215 UP
	0.3%	Xanthan
*S4*	1.1%	Glucopon 215 UP
*S5*	1.1%	Glucopon 215 UP
	0.3%	Xanthan


*Values are presented in percentages (volume/volume for hydrogen peroxide et weight/volume for Glucopon and Xanthan).*

### Test Materials

Tests were performed with coupons of polystyrene (petri dish polystyrene, Greiner Bio-one Co., Ltd., Frickenhausen, Germany) which is transparent and non-reactive with our disinfectant. The coupons were calibrated at 2.5 cm by 12 cm. Each coupon was sterilized overnight in a chemical bath containing hydrogen peroxide (ANIOS Co., Lille-Hellemmes, France), washed with 70% (v/v) ethanol to remove all disinfectant traces, and stored at 60°C in a sterile container until use.

### Disinfection Efficiency Test on Coupon

**FIGURE 1:**
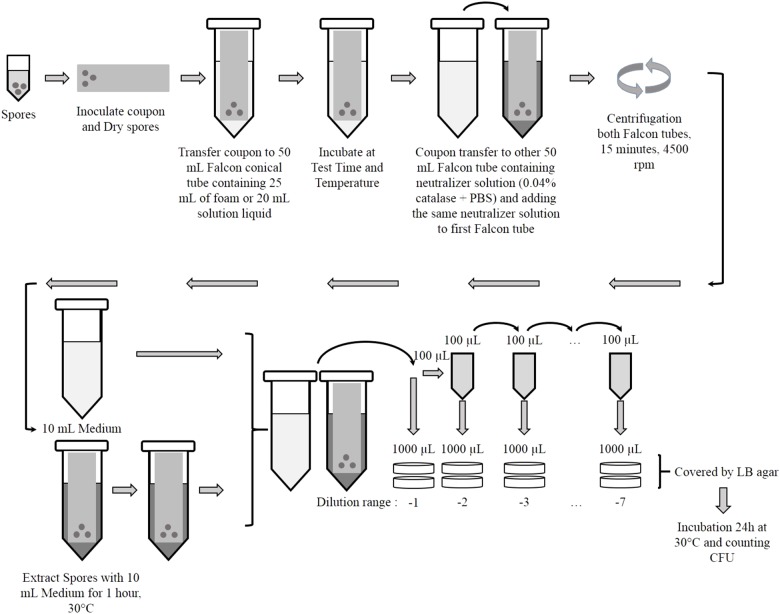
Step-by-step diagram of the foam and liquid solution decontamination method.

A bacterial suspension of *B. thuringiensis* spores was prepared in distilled water at a concentration of 10^9^ CFU/mL. An aliquot of 100 μL (10^8^ CFU/mL) was inoculated, in droplets, on the surface of each coupon with the help of a micropipette (**Figure 1**). After inoculation, the coupons were air dried in a biosafety cabinet (2 h maximum before use).

The inoculated coupons were then immersed in a 50 mL Falcon© tube containing approximately 25 mL of foam or 20 mL of liquid solution. The Falcon© tubes containing coupon were kept in vertical position at different controlled temperatures: 20°C inside a biosafety cabinet, 30°C inside a climatic chamber and 4°C in a refrigerator, for various times (5, 10, 15, 20, 25, and 35 min).

After the incubation each coupon was transferred into a new 50 mL Falcon© tube containing 10 mL of neutralizer solution of phosphate buffered saline (PBS) + 0.04% catalase (Sigma-Aldrich, St. Louis, Mo., United States) to stop the biocide activity. 20 mL of this neutralizer solution were added also to the Falcon© tubes containing only the foam (**Figure 1**).

To isolate the spores from the coupons, each tube was centrifuged at 4500 rpm for 15 min at 4°C. After removing the supernatant, the pellet containing spores was re-suspended with 10 mL of liquid LB. Each Falcon© tube containing coupon was incubated for 1 h at 30°C. Serial dilutions were plated for each Falcon© tube in LB media and covered with LB agar. Experiments were performed in duplicate. After drying, the plates were incubated for 18–24 h at 30°C and CFU were counted manually.

### Disinfection Efficiency Test Without Coupon

Bacterial suspension of *B. thuringiensis* at a concentration of 10^9^ CFU/mL, was used to test the decontamination efficiency of solutions without coupon. 100 μL droplets of this suspension were applied directly inside 20 mL of foam or 1 mL of liquid solution inside a 50 mL Falcon© at 20°C. After different contact times, these Falcons© were treated following the protocol described in **Figure 1**.

### Validation of Disinfectant Tests

Controls tests were carried out in parallel for each disinfectant test: (1) Blank control: coupons were not inoculated, but decontaminated as the inoculated ones. These coupons were used as control for issue related to cross-contamination during the test. (2) Positive control: each experiment included an assay where the solution did not contain disinfectant (*S4* and *S5* in **Table 1**). Protocol described in **Figure 1** was followed for both the foam and the liquid form without disinfectant. These tests indicated the amount of spores transferred in the liquid or in the foam by the coupon and the amount remained adhered on coupon. (3) Recovery of microorganisms: the same tests with solutions *S4* and *S5* were performed without coupon to evaluate the impact of the protocol on the recovery.

### Data Processing

Recovery rate and log_10_ reduction (LR) were determined at last three times in duplicate for each preparation. Recovery data were calculated for each preparation with and without coupons following the equation (1):

(1) $$Mean\;\%\;recovery=[Mean\;CFU_{pc}/CFU_{spike}]\times 100$$

where mean *CFU_pc_* is the mean of CFU recovered from at last three duplicate positive controls (pc) for each preparation with or without coupons, and *CFU_spike_* is the number of CFU spiked (inoculated) on the coupon, or inside the liquid or foam.

Decontamination efficacy for each treatment was calculated to measure the efficacy of the foam treatment in terms of the spore log_10_ reduction (LR). This was calculated by dividing the number of viable spores extracted after the decontamination tests by the number of spores originally spiked. The decontamination efficacy for biological agents was expressed in terms of a log reduction using the equation (2):

(2) $$Log\;Reduction\;(LR)=log(CFU_{t}/CFU_{spike})$$

where *CFU_t_* is the mean number of viable organisms recovered from either each coupon or the foam or liquid solution used for a test, after decontamination. Test coupons in which no CFU was recovered, were assigned a CFU count of 1, resulting in a log CFU of zero. Standard deviations were calculated from the mean results of the replicated experiments.

For each mean, the standard error of the mean (SEM) was calculated. *Student t* tests were performed using XLStat software (Addinsoft, Paris, France) to determine if two data sets of mean were significantly different from one another. A *p-value* <0.05 was considered to be significant.

### Evaluation of the Rate Constant for Inactivation and Arrhenius Law

In order to evaluate the effects of temperature on the decontamination efficacy for bacterial spores, the rate constant for inactivation *k* was deduced with a mathematic model based on first-order kinetics:

(3) $$dN/dt=\;-kN,\;\mathrm{or},\;\mathrm{in}\;\mathrm{the}\;\mathrm{integration}\;\mathrm{form},\;log(N/N_{0})=\;-kt$$

where *N_0_* is *CFU_control_* and *N* is *CFU_t_* at time *t* (including time zero). The slope of the linear regression of *log (N/N0)* versus time t is equal to *-k*.

In the Arrhenius equation, *k* is related to the inactivation energy of each decontamination parameter *E_a_* (kJ/mol^-1^) (Watanabe et al., 2003) by the equation:

(4) $$k=A_{0}e(-E_{a}/RT),\;\mathrm{or},\;\mathrm{in}\;\mathrm{the}\;\mathrm{integration}\;\mathrm{form},\;ln\;k=ln\;A_{0}-(E_{a}/RT)$$

where *k* is the rate constant for inactivation, which is a function of temperature *T, A_0_* is the frequency factor and *R* is the gas constant (8.314 J/mol.K^-1^).

A survival curve for each foam and liquid parameter was plotted for each temperature to measure *k*. For each foam and liquid parameter, the slope of the linear regression of *ln k* versus *1/T* enabled the determination of *E_a_/R* and *A_0_*.

## Results

### Determination of Spores Recovery Inside the Foam or Liquid Solutions in the Absence of Disinfectant

The spores recovery injected inside foam or liquid forms was determined with *S5* solution that did not contain disinfectant (**Table 1**). The mean recovery from foam form was higher than the liquid solution with respectively means of 8.03 ± 0.03 log and 7.70 ± 0.10 log, and statistically significant (*p*-value = 0.01; *t*-test on mean). This significant difference justifies the amount used in equation (1) (*CFU_spike_* = 10^8^ CFU/mL).

### Determination of the Spores Recovery in the Absence of Disinfectant on Coupon

The recovered amount of spores from coupon (adherent spores) was not statistically different when the coupon was in contact with foam or with liquid solution (*p*-value = 0.086). Similarly, the fraction of spores recovered inside foam or liquid (transferred spores) was not statistically different (*p*-value = 0.295). However, the total recovery of these controls was lower than for control tests without coupons. The recovered amount of spores from both adherent and transferred parts was 7.42 ± 0.15 log.

**FIGURE 2:**
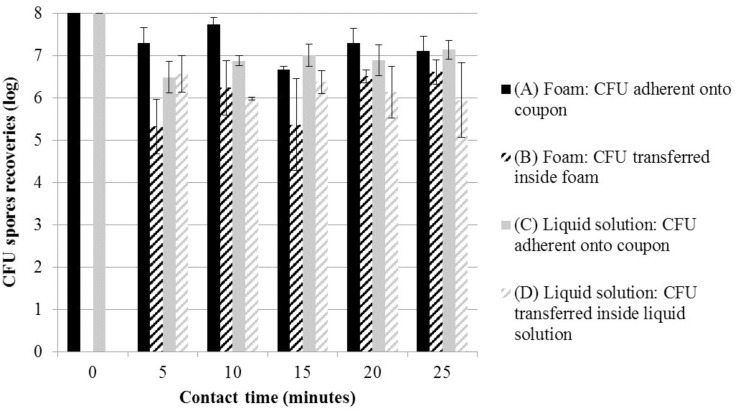
Mean log CFU (±SD) for tests without disinfectant (solution *S5* in Table 1); foam form: **(A)** adherent on coupons (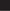) and **(B)** transferred inside foam (=); liquid solution: **(C)** adherent on coupons (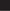) and **(D)** transferred inside liquid solution (=), at various contact times and room temperature. CFU, colony forming units.

A fraction of bacterial spores was rapidly transferred inside the foam or the liquid solution (**Figure 2**). Within 5 min, 5% average of the inoculated bacterial spores were transferred inside the liquid solution. For the transfer within foam, 10 min were needed to obtain 3% of the spores transferred. After 20 min, the quantity transferred from coupon to foam or liquid solution did not change and stabilized at 5%. Moreover, the total foam recoveries were always higher than the total liquid solution recoveries. After 25 min of treatment with our foam, 90% of spores still adhered on the surface. The surface was considered still contaminated with more than 10^7^ CFU. This value is considered dangerous for human health in the case of spores from bacterial pathogens.

### Decontamination Efficiency on Coupon at Room Temperature

**FIGURE 3:**
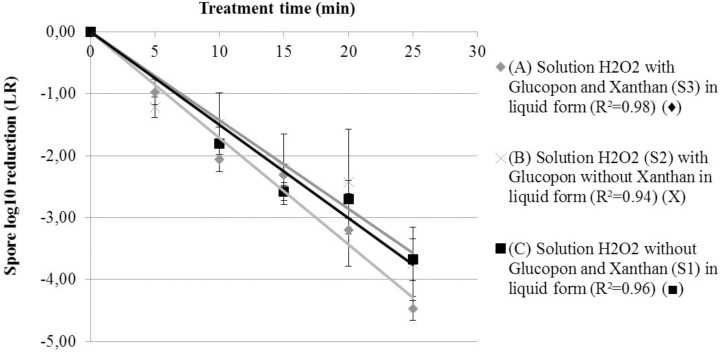
Spore survival adherent on coupon curves, at 293.15 K (20°C) at different contact times. **(A)** Solution H_2_O_2_ with Glucopon and Xanthan (*S3*) in liquid form (*R*^2^ = 0.98) (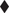). **(B)** Solution H_2_O_2_ (*S2*) with Glucopon without Xanthan in liquid form (*R*^2^ = 0.94) (X). **(C)** Solution H_2_O_2_ without Glucopon and Xanthan (*S1*) in liquid form (*R*^2^ = 0.96) (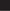). Unbroken lines for first-order kinetics.

To compare the decontamination efficiency on vertical surface between foam and liquid containing disinfectant, reaction inactivation kinetics were performed (*S1, S2*, and *S3* in **Table 1**). Experiments in liquid solutions enabled to characterize the effect of the foaming surfactant (Glucopon 215UP) and stabilizing agent (Xanthan) on the inactivation kinetic (LR) (**Figure 3**). After twenty-five minutes of contact time in a vertical position, the adhered spores on the coupons were not completely inactivated. The decontamination efficiency ranged from 3.68 log for the liquid solution without additive (*S1*), to 4.47 log for the liquid solution with all additives (*S3*). The decontamination kinetic of liquid solutions on spores adherent to the coupon can be seen in **Figure 3**, with the lines for first-order kinetic-model (Murdoch et al., 2016). The equation of these lines enabled the determination of the inactivation coefficients for each treatment at “room temperature” (equal 293.15 K and 20°C). The model did fit satisfactorily the result of each treatment, with R^2^ ranging from 0.94 to 0.98. Nevertheless, as can be seen in **Table 2**, no positive effect of Glucopon and Xanthan (*S3*) was revealed [difference not statistically significant compared to other treatments (*S1* and *S2*) (*p*-value >0.05)].

**Table 2:** The constant inactivation rate k for adherent spores on coupon, in accordance with different treatments at 293.15 K (20°C).

Treatment	Constant inactivation k (s^-1^)
Foam:H_2_O_2_ with Glucopon and Xanthan (*S3* in foam form)	0.1927
Foam: H_2_O_2_ with Glucopon and without Xanthan (*S2* in foam form)	0.1848
Liquid: H_2_O_2_ with Glucopon and Xanthan (*S3* in liquid form)	0.1715
Liquid: H_2_O_2_with Glucopon and without Xanthan (*S2* in liquid form)	0.1427
Liquid: H_2_O_2_ solution without Glucopon and without Xanthan (*S1*)	0.1503


**FIGURE 4:**
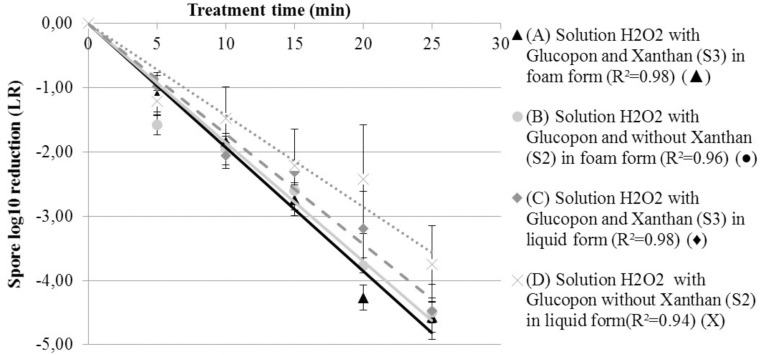
Spore survival adherent on coupon curves, at 293.15 K (20°C) at different contact times. **(A)** Solution H_2_O_2_ with Glucopon and Xanthan (*S3*) in foam form (*R*^2^ = 0.98) (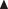). **(B)** Solution H_2_O_2_ with Glucopon and without Xanthan (*S2*) in foam form (*R*^2^ = 0.96) (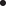). **(C)** Solution H_2_O_2_ with Glucopon and Xanthan (*S3*) in liquid form (*R*^2^ = 0.98) (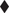). **(D)** Solution H_2_O_2_ with Glucopon and without Xanthan (*S2*) in liquid form (*R*^2^ = 0.94) (X). Unbroken lines for first-order kinetics for foam and broken lines for first-order kinetics for liquid solution.

**Figure 4** shows the LR as a function of time, for solutions containing hydrogen peroxide *S2* and *S3* in the both forms: liquid and foam. The same statistical model used for the liquid solution (**Figure 3**), was applied to determine the inactivation coefficients at “room temperature”. This model fitted correctly with the results, with R^2^ of 0.96 and 0.98 for the decontamination foam without (*S2*), and with stabilizing agent (*S3*) respectively. Using foam with an average of 5.5% of liquid fraction was enough to satisfactorily wet the surface with a liquid film that enables the spores to be inactivated. Also, the decontamination efficacy kinetic for both decontamination foams was almost similar and after twenty-five minutes of contact time, the mean LR was equal for both (4.5 log). However, after 45 min of contact time no CFU were retrieved on the surface of the coupons.

The inactivation coefficients (k s^-1^) of the spores transferred inside the foam were 0.209 with stabilizing agent (*S3*) and 0.288 without (*S2*). However, control tests with solutions *S4* and *S5* showed that only 5% of the bacterial spores were transferred within the foam after twenty-five minutes of contact time. The decontamination kinetic inside foam with (*S3*) and without (*S2*) the stabilizing agent was not statistically different (*p*-value >0.05). After twenty-five minutes, the decontamination was not completed in both foam fractions, but no CFU were retrieved after at least 35 min from the foam fractions even if they were found on the coupon (data not shown).

### Decontamination Efficiency at Different Temperatures

**FIGURE 5:**
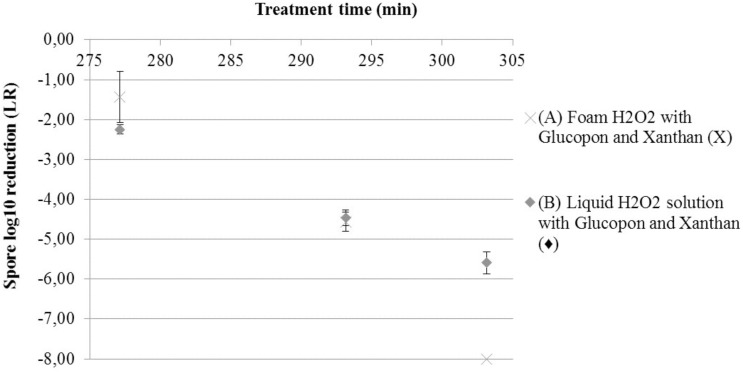
Spore survival adherent on coupon curves, at different temperatures after 25 min of contact time. **(A)** Solution H_2_O_2_ with Glucopon and Xanthan (*S3*) in foam form (X). **(B)** Solution H_2_O_2_ with Glucopon and Xanthan (*S3*) in liquid form (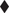) unbroken lines for foam and broken lines for liquid solution.

To determine the effect of the temperature on decontamination efficacy, the spore inactivation was assessed at three temperatures after twenty-five minutes of exposure. This efficacy was evaluated for solution *S3* in the foam and liquid form. At the lowest temperature (4°C = 277.15 K) the values of LR were very low and ranged from 1.44 to 2.25 LR (**Figure 5**). Cold temperatures drastically slowed down the decontamination kinetic on surface. On the contrary, a different behavior was found at higher temperature (30°C = 303.15 K). The LR results were different between the decontamination by foam and by liquid solution. For the foam, no bacterial spores were recovered on coupon after twenty-five minutes of contact time at 303.15 K, at the limit of detection. For the liquid solution, after the same contact time, the bacterial spore recovery was nearly 5.5 LR. The difference between both forms (foam and liquid solution) at 303.15 K was statistically significant (*p*-value >0.05).

### Activation Energy of Foams S2 and S3 (Arrhenius law)

**FIGURE 6:**
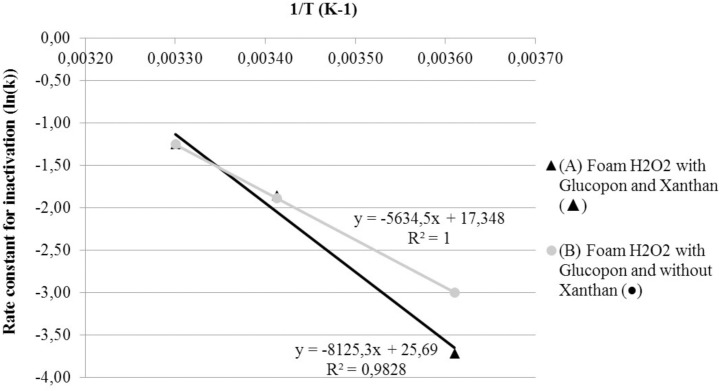
Effect of temperature on Arrhenius plots of the rate constant for spore inactivation. **(A)** Foam H_2_O_2_ with Glucopon and Xanthan (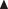). **(B)** Foam H_2_O_2_ with Glucopon and without Xanthan (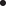). Unbroken lines for first-order kinetics for foam.

The activation energy was determined for foams *S2* and *S3*. For this comparison, CFU control (*CFU_pc_*) were used to calculate LR. To determine if results were fitted by the Arrhenius law, the reciprocal of absolute temperature was plotted as a function of the natural logarithm of the inactivation coefficient (**Figure 6**). The two foam curves were fitted by the Arrhenius law. The activation energies ranged from 46.85 KJ/mol^-1^ for foam without stabilizing agent (*S2*) to 67.55 KJ/mol^-1^ for foam with stabilizing agent (*S3*).

## Discussion

The efficiency to decontaminate *Bacillus thuringiensis* spore had already been proven for the described, newly patented, foam containing hydrogen peroxide. This efficiency was particularly tested on horizontal coupons (Faure et al., 2016). Hydrogen peroxide was selected as disinfectant for its antibacterial and sporicidal activity, and for its surface- and additive-friendly characteristics (Sagripanti and Bonifacino, 1999; Melly et al., 2002; Raber and Burklund, 2010; Eryilmaz et al., 2016). The hydrogen peroxide was known to be neutralized by catalase positive bacteria (McDonnell and Russell, 1999; Pottage et al., 2012). However, previous studies had shown that with high hydrogen peroxide concentration and a sufficient contact time this bacterial defense can be overwhelmed (Otter and French, 2009; Rios-Castillo et al., 2017). Recently in our laboratory, the Xanthan foam described in this study was already successfully tested on different bacteria used in French standard NF T 72-194: *Enterococcus hirae, Staphylococcus aureus, Salmonella typhimurium* and *Pseudomonas aeruginosa* (data not shown). Moreover, the sporicidal capacity of this foam was checked on horizontal surface on *Bacillus thuringiensis, Geobacillus stearothermophilus* (biological indicator for biosafety level 3 decontamination) and *Bacillus anthracis* (biological weapon). The foam enabled to reduce more than 6 log of these spores in 30 min of contact time on horizontal coupons (data not shown). Therefore, in this paper we investigate the decontamination behavior of this new foam solution with coupons in a vertical position.

Firstly, the formulation impact on the decontamination efficiency had been validated by experiments on liquid solution. The surfactant agent (Glucopon) enables the generation of a foam with an appropriate generator. The use of a stabilizing agent (Xanthan) in the foam allows the achievement of a sufficient contact time for decontamination in vertical position and by filling (Dame et al., 2005). Indeed, Dame et al. already showed the increase of a radiological decontamination foam stability and the duration of the wetting film on the wall. This effect is attributed to presence of Xanthan which delays the beginning of the drainage and after decreases the drainage kinetic. Moreover, our results proved that both products, Glucopon and Xanthan, have no negative impact on the decontamination efficiency of hydrogen peroxide. The comparison of results revealed that the disinfectant quantity in the foam with liquid fraction at 5.5%, is sufficient to obtain the same decontamination efficiency measured for the liquid solution. Thus, the wetting-film from a foam with a liquid fraction of average 5.5% is thick enough to wet the spore layer on the coupon to decontaminate. Furthermore, at this liquid fraction of the foam, only 5.5 L of liquid solution were needed to produce 100 L of foam. Therefore the quantity of effluent is also considerably reduced at the end of treatment compared to liquid solution treatment, for the same efficiency. Moreover, the Xanthan foam process is advantageous due to the sticking properties of the stabilizing agent in the foam. This one ensures a sufficient contact time in vertical position to neutralize all spores (spores reduction of 8 log for 45 min in this study). Thus, it is not necessary to apply the solution several times to ensure the manufacturer’s recommended contact times for vertical decontamination (Environmental Protection Agency, 2010; Edmonds et al., 2014). All these results in vertical position show that the foam could neutralized spores below 1 h and could be adapted to decontaminate rapidly sites where access is difficult, for example pipes, ceiling, walls with or without crevices, and areas behind equipment.

Most studies based on liquid and foam decontamination process were performed between 20 and 24°C (293–297 K) (Young and Setlow, 2004; Ryan et al., 2014) or “room temperature” (Rogers et al., 2005; Eryilmaz et al., 2016). For certain studies the experimental temperature has not been described (Andersen et al., 2006; Gonzalez et al., 2015). Whereas some papers advised to take this parameter into account for the evaluation of the decontamination efficiency (Russell, 1990; Edmonds et al., 2014). The influence of temperature on the decontamination efficiency of our foam is studied. Indeed, this foam could be used at different temperatures especially for the decontamination of outdoor sites (4°C = 277.15 K and 30°C = 303.15 K). The results show the decontamination kinetic of our hydrogen peroxide foam increases with raising temperatures. This observation is coherent with another foam preparation containing sodium hypochlorite (Guan et al., 2013) and other works on liquid hydrogen peroxide on horizontal surface (Hilgren et al., 2007). However, to our knowledge, this is the first time that a foam containing hydrogen peroxide is studied at these temperatures and in vertical surface. At 30°C the foam neutralized all bacterial spores after 25 min of contact time in a vertical position. However, the reaction inactivation kinetic is not linear with the increase of temperatures. At this temperature, the viscosity decrease speeds up and seems to produce a positive effect on the drainage inside the wetting film directly in contact with the spores. This effect enables the detachment of bacterial spores, which are neutralized inside the foam, as well as the disinfectant replenishment. This hydrodynamic effect is not found for liquid treatment, which is more static. This effect could explain the difference between foam and liquid solution efficiency when the temperature increases.

At 4°C the foam did not enable complete decontamination of the surface in 25 min. As the foam reaction follows the Arrhenius law, the decontamination kinetic can be predicted at any temperature. Therefore, it is possible to adapt the contact time between the foam and the contamination. A contact time of 2h30 at 4°C appears to be required to reach 8 log of reduction of spores. The adherent properties of the foam and the stabilizing agent ensure a sufficient wetting film thickness during this contact time. Thus, using the Arrhenius law, it is possible to adjust the contact time depending on the environmental temperature for the best use of the foam. For example, to decontaminate a vehicle in a garage, it is possible to calculate the contact time required, depending on the ambient temperature in different seasons (Andersen et al., 2006). In conclusion, the temperature plays a role in decontamination efficiency kinetic and should be taken into account before carrying out the assays, especially to compare the efficiency of different products (Wood et al., 2011).

In addition, this study demonstrated that bacterial spores were not only decontaminated on the coupon but also after being transferred inside the foam thanks to the wetting film. Previous studies have already discussed the complex issues of biological sampling and recovery from surfaces after testing (Rastogi et al., 2009; Tomasino et al., 2010; Edmonds et al., 2014). Tomasino et al. found that recovery from different materials ranged from approximately 20 to 70%. They studied the recovery from three different fractions. The first fraction remains on the sampling tool surfaces, i.e., tube. A second one was not retrieved, due to spore adhesion to the surface matrix. Finally, the third fraction corresponded to the spores retrieved from a coupon by sampling tools. These three fractions correspond well to those analyzed here by the recovery tests with and without coupons. However in our study, we measured also the amount of spores detached from the coupon and transferred within the foam. Even if there is a high rate of spore adhesion to the surface, the spore upper layer is transferred inside the foam by the wetting film. After 20 min, the fraction of spores transferred in the foam reached a maximum (**Figure 2**). However, all spores transferred into the foam are neutralized after 35 min with disinfectant. Finally, a recovery difference was also found between foam and liquid solutions. For the foam, the recovery is higher than the liquid solution. It would appear that the foam bubbles could facilitate the capture of hydrophobic spores, due to the attachment of the spores to the air-liquid interface. Consequently, the liquid effluents are safe after 35 min foam treatment, avoiding the contamination of other surfaces.

Major foam decontamination properties have been highlighted in this study. First, the formulation’s stability, wetting, and hydrodynamic properties improve the decontamination effects on adherent spore. Secondly, the foam process allows to inactivate the transferred spores into the foam. Finally, the temperature plays a key role on the decontamination kinetic and should be taken into account to compare the efficiency of disinfectants. This process was developed in context of CBRN-E Defense sector because it could treat large and complex shape facilities in case of biological attack or accident. But it could be useful also for other applications i.e., hospital, food, as can be seen from the many articles published on this topic (Benga et al., 2017; Mott et al., 2017; Szabo et al., 2017).

## Author Contributions

ELT, FG, NO, and SF designed and supervised the study. ELT, FG, and SF designed the experiments. ELT performed the experiments. ELT, FG, and SF analyzed the data. ELT, FG, NO, and SF wrote the manuscript.

## Conflict of Interest Statement

The authors declare that the research was conducted in the absence of any commercial or financial relationships that could be construed as a potential conflict of interest.
